# Microleakage Assessment of Different Preparation Techniques and Pit and Fissure Sealants in Permanent Molars

**DOI:** 10.7759/cureus.50382

**Published:** 2023-12-12

**Authors:** Madeeha Bangash, Mashal Humayun, Miraat Anser, Farooq Ahmad Chaudhary, Bilal Arjumand, Hamad Mohammad Alharkan

**Affiliations:** 1 Department of Paediatric Dentistry, Rehman College of Dentistry, Peshawar, PAK; 2 School of Dentistry, Shaheed Zulfiqar Ali Bhutto Medical University, Islamabad, PAK; 3 Department of Conservative Dental Sciences and Endodontics, College of Dentistry, Qassim University, Buraydah, SAU

**Keywords:** penetration, prevention dentistry, etching, prophylaxis paste, microabrasion

## Abstract

Background: This study aimed to evaluate the predisposition of microleakage in permanent molar teeth following different preparation techniques for pits and fissure sealants.

Methods: In this cross-sectional analytical study, a dye penetration method was employed to evaluate microleakage in dental restorations. A total of 104 extracted molars were randomly assigned into two groups and further subdivided into two subgroups based on a class of sealant (filled/unfilled resin) containing 26 teeth each. Teeth in one group were prepared by a conventional method using pumice and acid etching with 37% phosphoric acid, and teeth in the other group were prepared with a 1/4-round carbide bur in a low-speed handpiece and then acid etched. In each group, 26 teeth were sealed with a filled sealant, and 26 teeth with an unfilled sealant. The chi-square test was used for the comparison of microleakage between the groups.

Results: Unfilled sealants prepared with bur preparation showed the lowest degree of microleakage. The greater number of teeth (17) showed no microleakage in the group of teeth prepared using the bur preparation technique. Furthermore, in dye penetration analysis, the subgroup of teeth that were filled with unfilled sealants showed a greater number of teeth (57.6%), with zero penetration, and the dye penetration scores were statistically significant among the groups with different preparation techniques (p=0.002).

Conclusion: Teeth whose pits and fissures were prepared through 1/4 carbide bur and filled with unfilled pits and fissure sealants show less microleakage than those prepared with the conventional method and filled with filled sealants. Therefore, the choice of surface preparation technique for pit and fissure sealants can influence the effectiveness of the sealant in preventing microleakage.

## Introduction

Dental caries is one of the most prevalent multifactorial adolescent diseases, which is modulated by food plan and teeth morphology [1]. The preventive measures showed greater effectiveness on the smooth surfaces of the teeth while caries on occlusal surfaces remain a problem. The occlusal or so-called hidden caries usually get missed during visual examination because, in many cases, it lacks the typical cavity design in enamel, and a radiograph and a fissure biopsy are needed for its detection [2].

The high frequency of occlusal caries has been reported in various studies due to the specific anatomy of occlusal surfaces that facilitate the retention of food remnants and bacteria [3]. Molars and premolars are particularly susceptible to caries due to the morphology of their occlusal surfaces. The varied pit and fissure shapes, classified as (Y, IK, V, U, and I), along with crevices and irregularities, create areas where food residues and bacteria become mechanically trapped [4]. The vulnerability of these teeth is associated with their unique occlusal morphology. Research indicates that fissure caries account for approximately 90% of caries in permanent posterior teeth and 44% in primary teeth. Specifically, the deep pits and fissures provide flourishing surroundings for the oral microorganisms to thrive and convert the carbohydrates into acids, which form the basis for the demineralization of the enamel [5].

A group of experts assembled by the American Dental Association (ADA), Council on Scientific Affairs (CSA), and American Academy of Pediatric Dentistry has concluded that pit and fissure sealants demonstrate greater effectiveness in halting and preventing the advancement of pit-and-fissure occlusal caries lesions in both primary and permanent molars among children and adolescents [6]. This efficacy surpasses the outcomes observed with fluoride varnish application and the absence of sealant use [7].

Sealing caries-prone sites is essential for effective caries prevention. Occlusal sealing significantly decreases the risk of caries compared to unsealed teeth. The pit and fissure sealants are applied on the occlusal pits and fissures of caries-susceptible teeth, which leads to the formation of a micromechanically bonded protective layer that blocks the access of caries-producing bacteria [8]. The introduction of the dental pit and fissure sealant marked the start of a major revolution in preventive dentistry [9].

Biocompatibility and resistance to abrasion and wear are the important properties of good pit and fissure sealants. However, many other factors also contribute to the success and effectiveness of pit and fissure sealants, such as adhering to the teeth surface, retention capability, and marginal sealing ability to prevent marginal leakages [7,9]. The preparation of pits and fissure surfaces before applying sealants is important to achieve the desired above-mentioned properties. In vitro studies can help in determining the effective preparation techniques to maximize the marginal sealing ability and limit the micro leakages. Therefore, in this in vitro, the objective is to assess the predisposition of microleakage in permanent molars following different preparation techniques for pits and fissure sealants.

## Materials and methods

A total of 104 extracted human maxillary and mandibular molars were included in this in vitro analytical study using the convenience sampling method. The sample size was calculated using G-power with an effect size of 0.3, α-error of 0.05, and Power of 0.95. Teeth with intact occlusal surfaces were included in the study, while those teeth with developmental defects, carious, hypo mineralized, broken, and fractured occlusal surfaces were excluded from this study. The approval of this study was taken from the Ethical Review Board of Shaheed Zulfiqar Ali Bhutto Medical University, Islamabad, Pakistan (Ref. no. 2022/021/87F). These extracted teeth were cleaned and then stored in a thymol solution to prevent bacterial growth. The teeth were randomly divided into two groups each containing 52 teeth.

Group 1 teeth were prepared using a traditional method of acid etch technique, followed by cleaning using a polishing paste [10]. The etching of the surface was performed by applying 35% phosphoric acid gel for 30 seconds and afterward rinsing and drying. In Group 2, pits and fissures were prepared using 1/4-round carbide bur in a slow-speed handpiece, and then acid etched similarly as Group 1 teeth. Teeth of both groups were then segmented into two subgroups, Subgroups 1A and 1B and Subgroups 2A and 2B, assigned with 26 teeth in each subgroup. The pit and fissure area of teeth in Subgroups 1A and 2A were packed with unfilled sealants specifically using UltraSeal XT hydro. Its composition comprises triethylene glycol dimethacrylate (TEGDMA), diurethane dimethacrylate (DUDMA), and methacrylic acid (MAA), while the pit and fissure area of teeth in Subgroups 1B and 2B were applied with filled sealant utilizing Nexcompo Flow. The composition of Nexcompo Flow comprises Bis-GMA, Bis-EMA, urethane dimethacrylate, and triethylene glycol dimehacrylate. Afterward, the teeth were stored in artificial saliva that was not renewed, organized through the technique described in the literature [11], for seven days in an incubator at 38 °C in order to create a surrounding that is in concord with the natural oral environment. Before testing, the teeth were thermos-cycled for 500 cycles at 6 °C and 47 °C for 30 seconds and dried, apices were then covered with sticky wax, and the surface of each specimen was covered with two layers of nail varnish, leaving a 1-mm window around the sealant [10]. Following the application of sealants, the teeth were further dipped in a 1% solution of methylene blue for 24 hour at 37 °C and cleaned with tap water. Each tooth was sectioned buccolingual direction in the mesial and distal surfaces using a water-cooled, slow-speed diamond disc saw.

**Figure 1:**
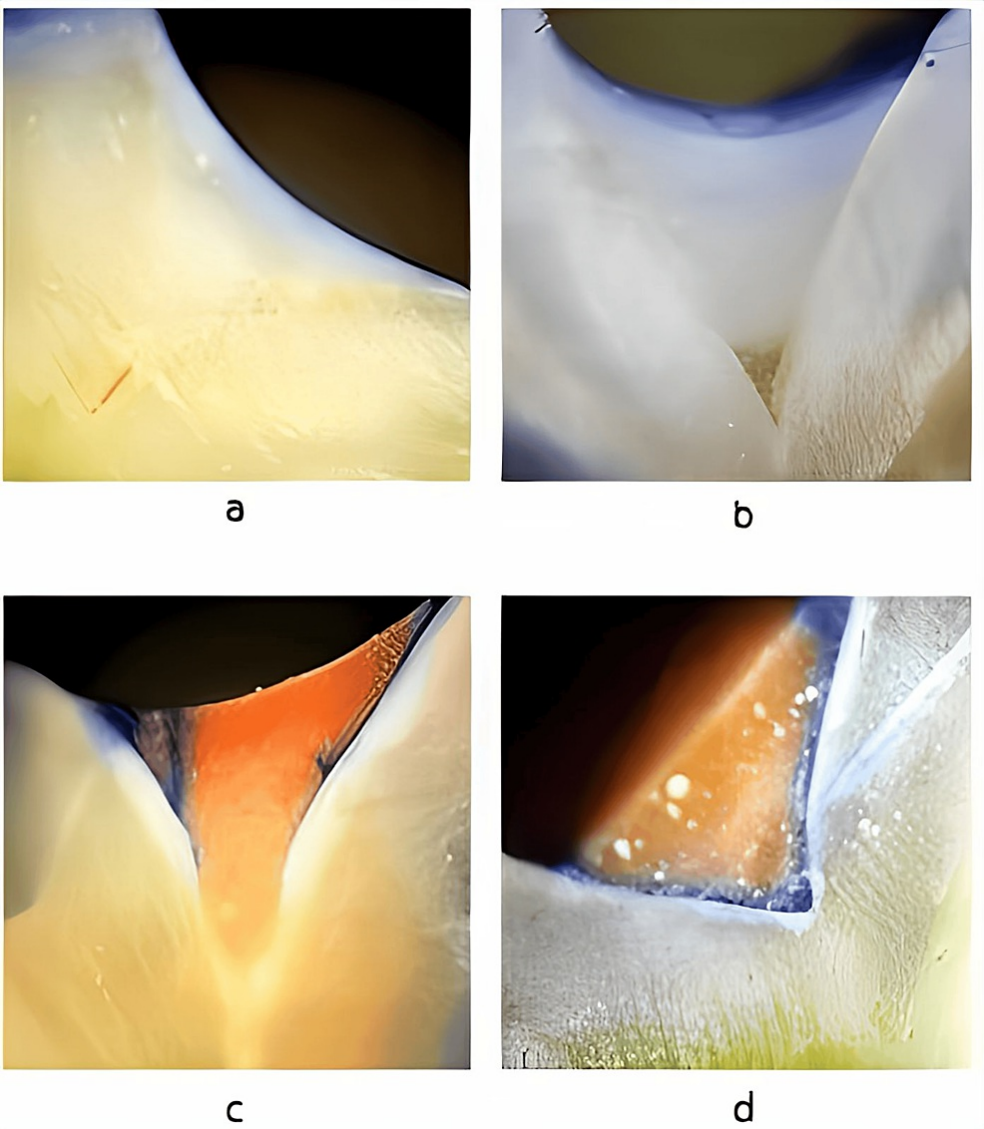
Photographs of the sample with a scoring system: (a) Score 0, (b) Score 1, (c) Score 2, and (d) Score 3.

For assessing dye penetration, each section of the specimens was tested at four different points (central pit, buccal cusp, palatal/lingual cusp, mesial/distal marginal ridge) under x20 magnification of a stereomicroscope by a single observer's using a method defined by Overbo et al. [12]. The calibration of the observer was done through thorough training before the study to ensure its capability to clearly understand the study objectives, measurement tools, specific criteria, and protocols to follow. The method of scoring is as follows (Figure 1).

1. Score 0-No dye penetration

2. Score 1-Dye penetration restricted to the outer half of the sealant

3. Score 2-Dye penetration to the inner half of the sealant

4. Score 3-Dye penetration into an underlying fissure

Statistical analysis was performed using Statistical Product and Service Solutions (SPSS, version 23) (IBM SPSS Statistics for Windows, Armonk, NY). Descriptive statistics were performed for means, counts, standard deviations, and categorical variables were reported in terms of frequencies and percentages. The chi-square test was used for the comparison of microleakage between the groups and analysis of the difference between an unfilled and filled sealant leakage. A p-value of ≤0.05 was considered significant for all tests.

## Results

Table 1 shows dye penetration scores among different groups according to the preparation techniques used. In Subgroup 1A, four 15%) teeth showed zero penetration, while in Sub-group 1B, one (3.8%) showed zero penetration. In Subgroup 2A, 15 (57.6%) teeth showed zero penetration, while in 2B, only two (7.6%) teeth showed zero penetration (p<0.05).

**Table 1:** Dye penetration scores among different groups according to the preparation technique. * Statistically significant (p<0.05)

Groups		Dye penetration score (n)(%)				Mean (SD)	P-value
		0	1	2	3		
Group 1 (Conventional technique)	1A	4(15%)	5(19%)	4(15%)	13(50%)	2.00 (1.16)	0.05*
	1B	1(3.8%)	1(3.8%)	3(11.5%)	21(80%)	2.69 (0.73)	
Group 2 (Bur preparation)	2A	15(57.6%)	11(42%)	0(0%)	0(0%)	0.42 (0.50)	0.05*
	2B	2(7.6%)	14(53%)	6(23%)	4(15%)	1.46 (0.85)	

**Figure 2:**
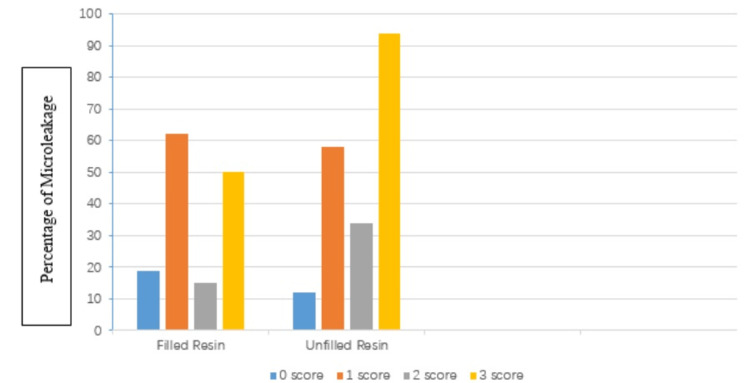
Comparison of the penetration of dye by the type of sealant used.

Figure 2 shows dye penetration according to the type of sealant material used. Regardless of the tooth preparation method, the teeth with unfilled sealant reported significantly less microleakage (p<0.05) compared to filled sealants.

## Discussion

It is important to evaluate the marginal microleakages of the fissure sealants as it shows the sealant's capacity to avoid bacterial infiltration through microleakages and decrease the chances of developing secondary caries [13]. The success of sealant mainly depends upon the etching techniques, duration, and acid used for etching; surface properties and anatomy of enamel; and its preparation method. In vitro studies on sealants are important to test new technologies, materials, and methods. The dye penetration technique to evaluate the microleakage in sealant is one of the preferred and widely used methods due to its nontoxic nature, low cost, and easy availability [14,15]. In this study, this dye penetration technique was used to evaluate the microleakage of the sealant with different preparation methods. 

In this study, the sealants showed much less microleakage in those teeth that were shaped by using bur and accompanied by acid etching, instead of traditional unprepared tooth surfaces. Preparation of the fissures by bur results in greater adaptation, good apparent texture, and deeper penetration due to the widening and deepening of the pits and fissures and the elimination of organic material, plaque, and a very thin layer of prism-less enamel [16]. Previous studies have revealed that failure of the fissure sealant is usually attributed to operator skill, patient cooperation, saliva contamination during the procedure, and change in the structure of enamel after acid etching and resin maneuvering [17]. In consistence with our findings, a study by Geiger et al. reported that the quality of pits and fissure sealants can be enhanced by mechanical preparation of the fissures as compared to non-prepared fissures even though Geiger et al. used bur with high-speed handpiece, while in this study, bur was used with a slow-speed handpiece. In the same study, it was shown that preparation with tapered diamond bur exhibited better results, rather than round carbide bur, while avoiding overfilling of the fissures [18]. In another study, unprepared-filled fissures showed greater sealant loss (6.25%) than sealant in prepared fissures with diamond bur [19]. The mechanical preparation with bur facilitates the retention of sealant in fissures due to an increase in the surface area.

A study conducted by Joshi et al. [20] in 2019 revealed compelling results. Following a 12-month observational period, the mean dental caries scores, assessed using the decay, missing, and filled teeth (DMFT) index, and the incidence of caries in deciduous second molars were significantly elevated in the control group when contrasted with the study groups that received pit and fissure sealants [20]. This underscores the potential efficacy of pit and fissure sealants in mitigating dental caries in primary teeth, emphasizing their importance as a preventive measure.

In this study, it was observed that unfilled resin materials used as fissure sealants displayed better retention and enactment as compared to filled resins, and this finding is consistence with the findings of other studies [21,22]. The higher load of the filler content in filled resins makes it more viscous, thereby reducing its adaptation and penetration to the bottom of deep pits and fissures as compared to hydrous unfilled resins [23]. A study by Autio-Gold showed that caries development was slightly higher in an unfilled resin material as compared to medium-filled resin-based sealants after 18 months of testing [24]. This difference can be attributed to the lower abrasive resistance of unfilled resin-based sealants. The literature showed inconsistent results while comparing microleakage among filled and unfilled resin materials used as fissure sealants. A study by Singh et al. reported no statistically significant difference in microleakage between the filled and unfilled resins [25]. Few previous studies have mentioned that saliva contamination can contribute to sealant microleakage and integration of fluoride into the sealant matrix can have a profound impact on sealant retention [22,26]. The plausible reason for the variation in the results of previous studies can be due to the use of different testing materials in different studies. In another study, teeth setup using pumice prophylaxis demonstrated less microleakage than air abrasion [27]. When using the pumice prophylaxis method for penetration of sealants into pits and fissures, only the inclined cuspal planes were clean, leaving pits and fissures packed with debris, residual material deposits, and air entrapment that notably affect sealant penetration into pits and fissures [27,28]. Studies have shown that increasing the surface area results in a thicker filling layer, contributing to enhanced wear resistance. A study by Eliacik et al. reported that mechanical preparation, involving the enlargement and deepening of fissures, leads to increased bonding strength [29]. In the realm of minimally invasive dentistry, resin infiltration has emerged as a novel approach.

The variation in the instrument, testing substance, and type of dye used contributes to the inconsistency of results and makes a comparison between studies difficult. Methylene blue, silver nitrate, and basic fuchsin are some of the dyes used in previous studies with different molecular sizes and affect their penetration into the pit and fissure sealants [17,19]. A large variation also exists among dentists regarding the use of various instruments, as well as the extent of cavity preparation for fissure sealants, which has a direct impact on the retention and success rate of the said procedure [7,21]. Seemingly, the majority of dentists prefer a light sweep with bur, instead of removing the chalkiness completely. More elaborated work needs to be done to establish a correlation between the efficacy, clinical success, and penetration of the dyes into the fissure sealants. To acquire more meaningful results from microleakage studies, it should involve replicating in vitro studies with in vivo studies, which will help in establishing the sealing abilities of the materials.

There are a few limitations to this study. Firstly, the application of sealants to teeth occurred in a controlled environment without the presence of saliva, which may differ significantly from the conditions in the oral cavity. Saliva can introduce variables such as varying levels of moisture, potential contamination, and other factors that may influence the performance of fissure sealants. Furthermore, the performance of fissure sealants may vary in different environments due to factors such as fissure morphology and preparation, acid etching of the enamel surface, adhesive application, and contamination of fissure surfaces. Secondly, the application of sealant procedure is technique sensitive, and operator skill as well as its dependent variables on the effectiveness of fissure sealants can affect its outcome. Future long-term follow-up studies should be carried out to assess the durability and longevity of sealant effectiveness, along with evaluation of the practical applicability of the findings in a clinical setting that involves factors such as patient comfort, time efficiency, and cost-effectiveness to ensure that recommended techniques and sealant types are feasible and beneficial in dental practice. Furthermore, a comprehensive comparative analysis of various types of sealants beyond those examined in the initial study should be carried out and incorporation of advanced imaging techniques, such as micro-CT scans, to provide detailed insights into the microstructure of sealed teeth. Lastly, a multidisciplinary collaboration between dental researchers and professionals from related disciplines, such as materials science and engineering, should be done as this interdisciplinary approach can foster the development of innovative sealant materials and techniques with improved properties.

## Conclusions

The conclusions drawn from this study emphasize the significance of surface preparation for the success of sealant application. Specifically, tooth surfaces prepared with a 1/4 round carbide bur demonstrated lower levels of microleakage when compared to those prepared using conventional methods. This suggests that the choice of surface preparation technique can influence the effectiveness of the sealant in preventing microleakage. Furthermore, the type of sealant used played a role in the observed outcomes. Teeth filled with unfilled sealants exhibited reduced microleakage in comparison to those teeth filled with filled sealants. This implies that the composition of the sealant, particularly whether it is filled or unfilled, contributes to its ability to create a more effective barrier against microleakage. Therefore, this study emphasizes the importance of both the surface preparation techniques and the composition of sealants in achieving successful outcomes.
